# Arzanol Inhibits Human Dihydroorotate Dehydrogenase and Shows Antiviral Activity

**DOI:** 10.1021/acs.jnatprod.5c00887

**Published:** 2025-10-27

**Authors:** Marta Alberti, Martina Tamburello, Stefano Salamone, Giorgio Gallinella, Cinzia Sanna, Giovanni Battista Appendino, Marco L. Lolli, Alberto Massarotti, Federica Pollastro, Riccardo Miggiano

**Affiliations:** † Department of Pharmaceutical Sciences, 19050 University of Piemonte Orientale, Via G. Bovio 6, 28100 Novara, Italy; ‡ Department of Medical and Surgical Sciences, 9296 Alma Mater Studiorum University of Bologna, Via Massarenti 9, 40138 Bologna, Italy; § Department of Pharmacy and Biotechnology, Alma Mater Studiorum University of Bologna, Via Massarenti 9, 40138 Bologna, Italy; ∥ Department of Life and Environmental Sciences, 3111 University of Cagliari, Via Sant’Ignazio da Laconi 13, 09123 Cagliari, Italy; ⊥ Department of Sciences and Drug Technology, 9314 University of Torino, Via P. Giuria 9, 10125 Torino, Italy

## Abstract

Human dihydroorotate dehydrogenase (*h*DHODH), catalyzing the rate-limiting step of the pyrimidine biosynthesis pathway (PBP), is a drug target extensively investigated for various diseases including cancer, autoimmune disorders, and viral infections. We present evidence that the heterodimeric phloroglucinylpyrone arzanol potently inhibits *h*DHODH by competing with ubiquinone for binding to the *lipophilic patch* (LP) of the enzyme. Co-crystallization experiments on the enzyme–arzanol complex provided further insights into the binding pocket of *h*DHODH, revealing detailed features that could inspire the design of innovative inhibitors. The cellular translation of these enzymatic and biochemical data was validated in antiviral assays on SARS-CoV-2 infected cells. Taken together, these results exemplify the potential of natural products to explore novel areas of the protein druggable space and provide information relevant to multiple critical areas of drug discovery.

## Graphical abstract

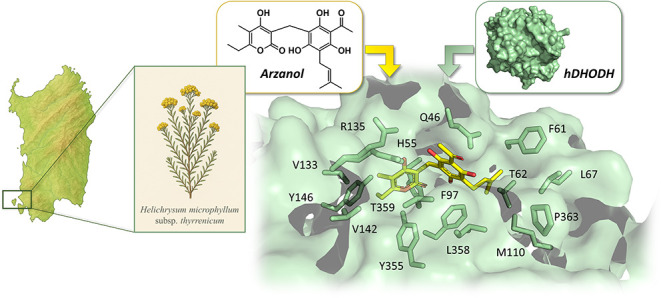

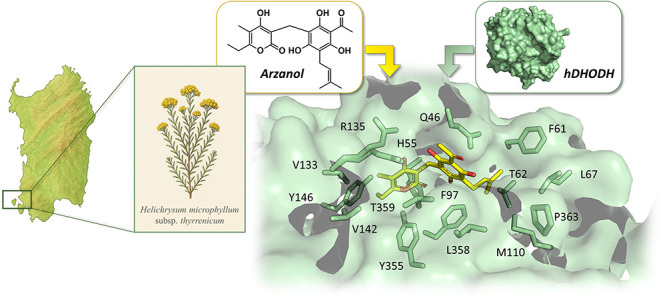

Human dihydroorotate dehydrogenase (*h*DHODH) plays a critical role in the *de novo* pyrimidine biosynthesis pathway (PBP), catalyzing the oxidation of dihydroorotate (DHO) into orotate (ORO) through a redox ping-pong mechanism. This reaction is coupled with the reduction of flavin mononucleotide (FMN), which is subsequently restored by coenzyme Q10 (CoQ10), also known as ubiquinone (see the catalytic reaction in Scheme 1).

**Scheme 1:**
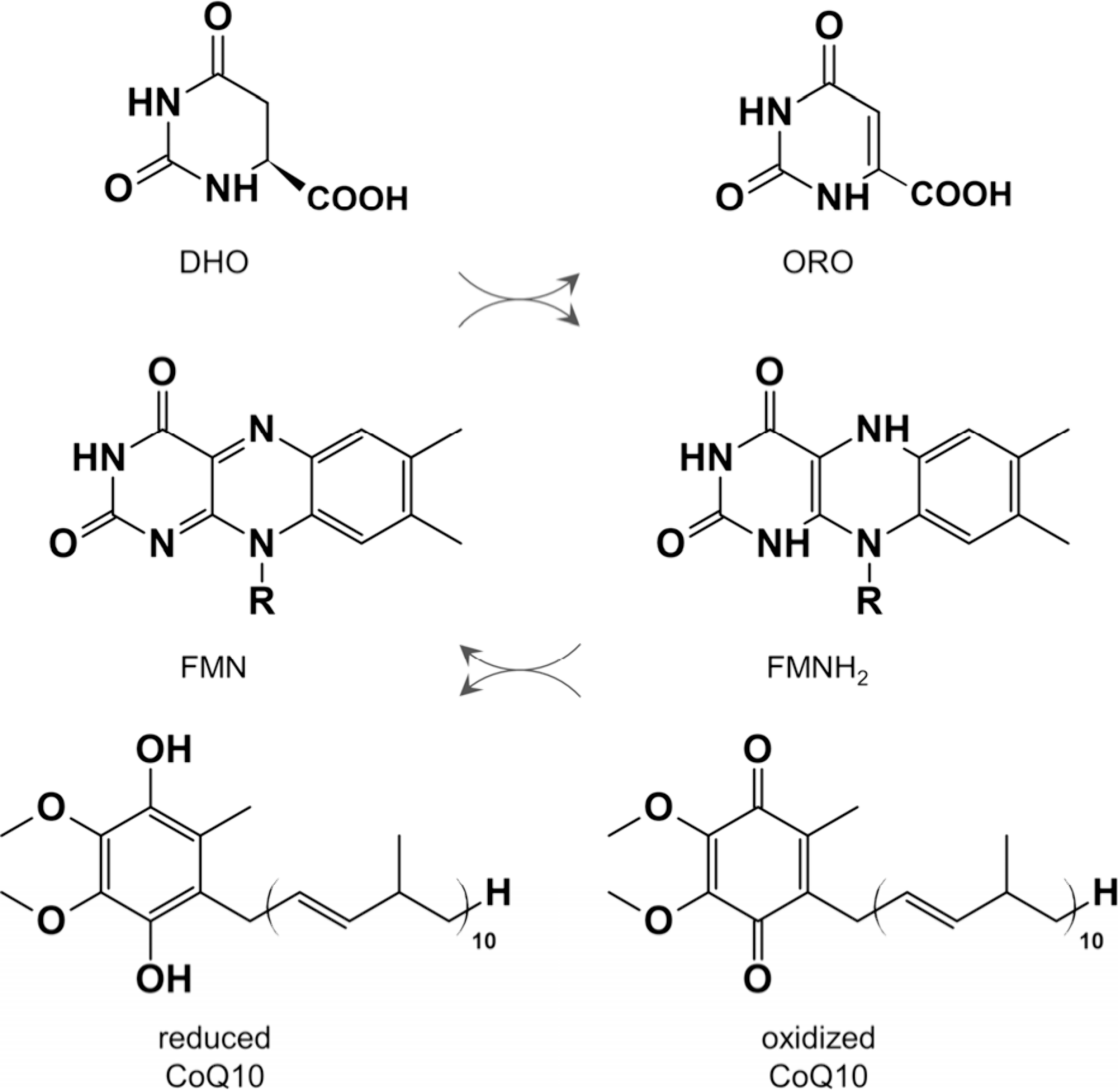
Ping-Pong Mechanism Reaction Catalyzed by *h*DHODH^a^

a The substrate DHO is oxidized to ORO through the concomitant reduction of FMN cofactor to FMNH_2_, which CoQ10 subsequently restores.

*h*DHODH is a validated target for the treatment of autoimmune diseases,^1^ including multiple sclerosis,^2^ lupus,^3^ and rheumatoid arthritis,^4^ and it has also been associated with the treatment of both solid^5,6^ and liquid^7^ cancers. On the other hand, the strong metabolic requirement of virus-infected cells makes the inhibition of *h*DHODH interesting also as a host-targeting antiviral (HTA) strategy,^8–14^ since depletion of pyrimidine synthesis limits not only viral replication but also the activation of innate immune responses and an excessive pro-inflammatory cytokine production (cytokine storms).^15^ Considering these insights, *h*DHODH inhibitors are under investigation in phase I/II clinical trials for the treatment of various viral infections (Table 1). Together with the involved clinical candidates, the advanced preclinical candidate MEDS433,^16^ designed by some of the authors, has also been included.

**Table 1:**
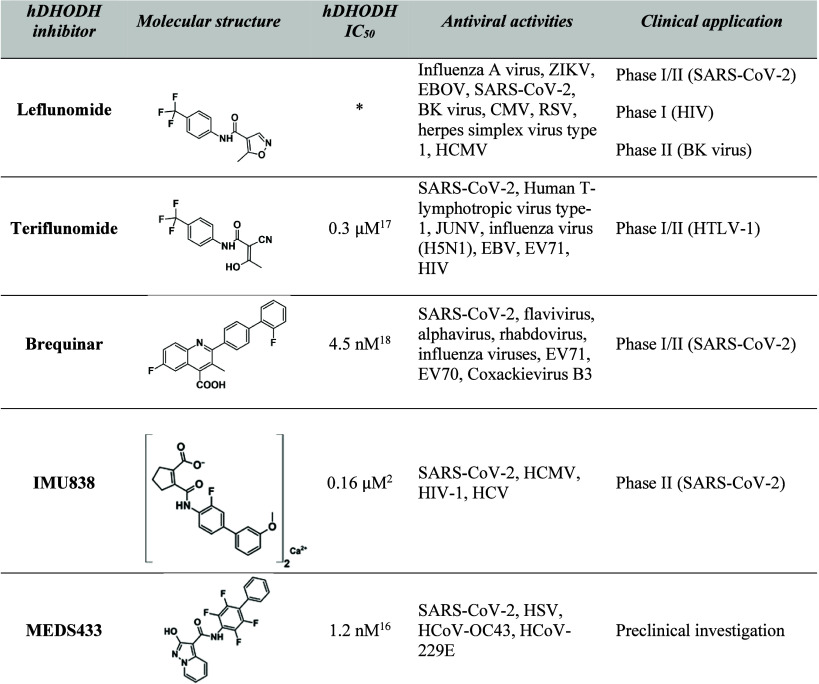
Antiviral Activities and Clinical Applications of Known *h*DHODH Inhibitors^a^ ^17,18^

a *: *In vivo* metabolized into teriflunomide. Abbreviations: CMV, cytomegalovirus; EBV, Epstein–Barr virus; EV, enterovirus; HCoV, human coronavirus; HSV, herpes simplex virus; JUNV, Junin virus; SARS-CoV-2, severe acute respiratory syndrome coronavirus.^1,19–22^

Moreover, DHODH has garnered considerable attention as a therapeutic target in diverse fields. Indeed, the targeting of DHODH has been investigated for its potential benefits against pathogenic bacteria^23–25^ and human parasites^26–28^ and as candidates for herbicides^29–32^ due to its unique biochemical properties and the essentiality of pyrimidine nucleotides for both microbial and plant proliferation.

In class 2 DHODHs, to which the human isoform belongs, an extended *N*-terminal domain anchors the enzyme to the inner mitochondrial membrane, forming a hydrophobic tunnel, the so-called *lipophilic patch* (LP), that harbors CoQ10 toward FMN. Acting as CoQ10 mimics,^33,34^ potent *h*DHODH inhibitors like IMU838 and brequinar, as well as MEDS433 itself, are lipophilic molecules characterized by an acidic polar head, able to interact with the LP also in polar *subsite 2* (R135 and Q46), described by Baumgartner et al.,^35^ close to FMN.

Arzanol, a phloroglucinylpyrone bearing a polyoxygenated hexasubstituted phenyl moiety and representing a major constituent of the Mediterranean plant *H. microphyllum* (Willd.) Cambess. subsp. *thyrrenicum* Bacch. Brullo and Giusso^36^ (Figure 1), has been reported in the literature to exhibit a broad spectrum of pharmacological activities, including anti-HIV,^36^ anti-inflammatory,^37,38^ and antioxidant^38–41^ effects. Its antiviral,^42^ antimicrobial,^43^ and antifungal^44^ properties also highlight its potential as a therapeutic agent for infectious diseases. Despite this wide range of biological effects, the molecular mechanisms underlying arzanol’s activity remain poorly characterized due to limited investigation into its specific molecular targets.

**Figure 1:**
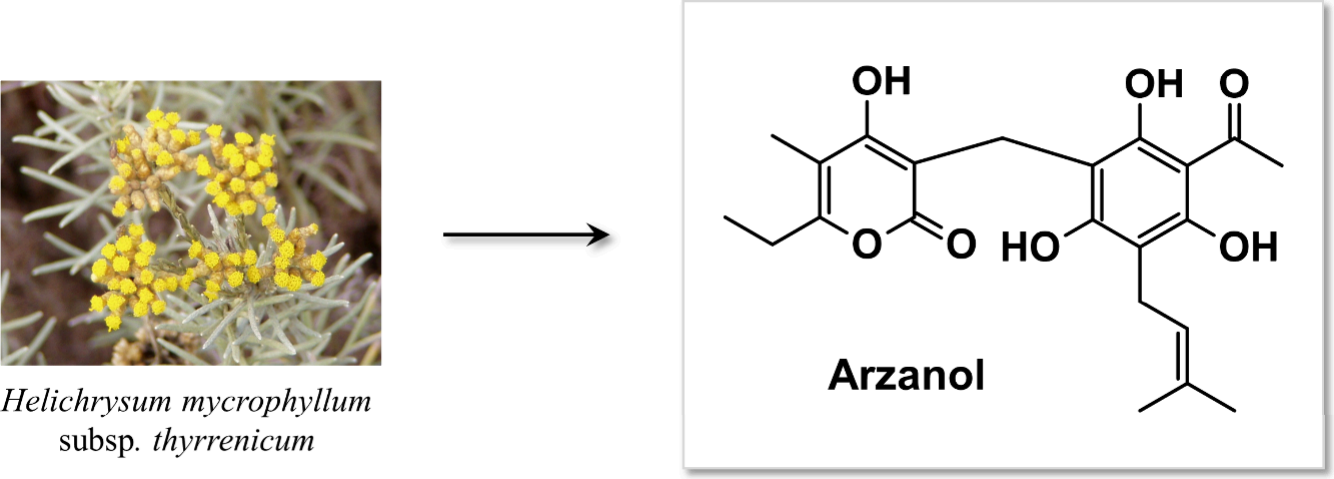
Chemical structure of arzanol, a natural compound extracted from *Helichrysum microphyllum* subsp. *thyrrenicum*. Image of *Helichrysum microphyllum* subsp. *tyrrhenicum*, used under a Creative Commons CC BY-SA 3.0 license. Photo by Júlio Reis, sourced from Wikipedia.

The arzanol structure is characterized by the presence of two polysubstituted aromatic rings (Ring A and Ring B, Figure 2) connected by a methylene bridge. At first glance, its structure appears to show a certain similarity with the aryl ring of the reduced form of CoQ10. Moving closer to ring A, similarly to the ascorbic acid itself (p*K*_a_ 4.36),^45^ a vinylogous carboxylic acid is present, characterized by a vinyl unit connecting the two groups that define the acidity: the carbonyl C=O and the OH moiety. Different from CoQ10 itself, the hydroxyl group on ring A, particularly acidic due to electron conjugation with an estimated p*K*_a_ of approximately 4, could be postulated to be deprotonated at physiological pH, a property shared with potent *h*DHODH inhibitors such as brequinar itself. Moving to ring B, the presence of lipophilic moieties could mimic the CoQ10 lipophilic tail in the interaction with *h*DHODH *subsite 1* (A54, F61, L67, F97, M110, and L358).

**Figure 2:**
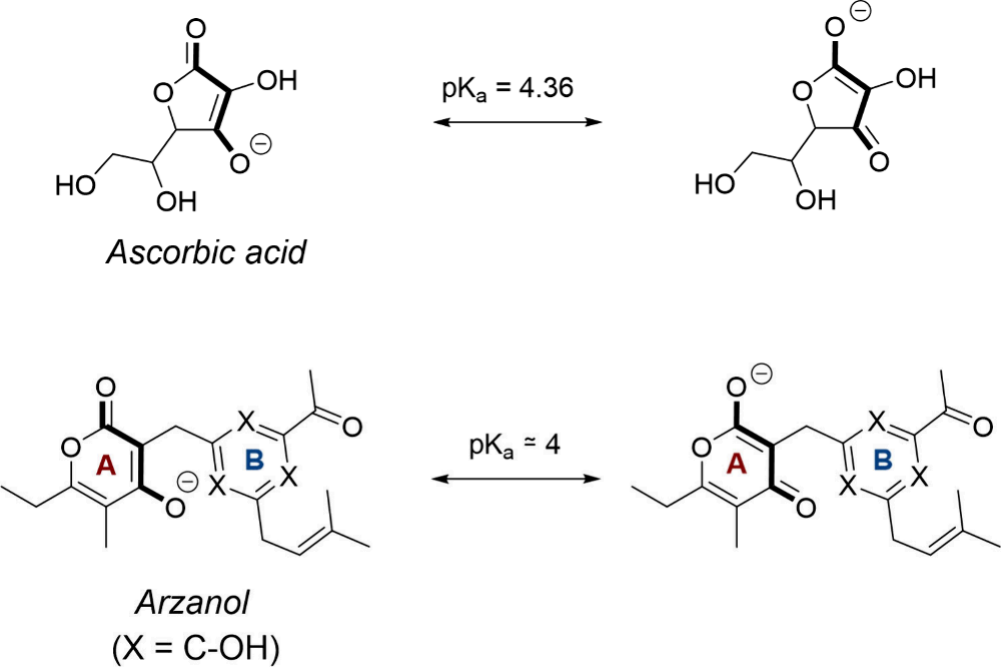
Structures and reactivities of ascorbic acid and arzanol, highlighting the delocalization of the negative charge within their vinylogous carboxylic acid groups. p*K*_a_ values are indicated in relation to ionization at a physiological pH.

The scaffold’s similarity between CoQ10 and arzanol, coupled with its antiviral and immunomodulatory activity profile, provided a rationale for us to evaluate its inhibitory activity on *h*DHODH, a target capable of potentially rationalizing the dual biological profile of the natural product. In this study, we presented evidence that arzanol inhibits *h*DHODH by competing with CoQ10 for binding to the LP. A series of cocrystallization experiments revealed the experimental binding mode of arzanol, which is distinct from that of the above-mentioned known inhibitors. Furthermore, antiviral assays conducted on the SARS-CoV-2 strain highlighted the potential application of arzanol as a lead compound in the development of novel HTA therapeutic agents.

## Results and Discussion

A validated enzyme preparation was used to screen a library of natural products, which were rationally selected for the presence of a quinone moiety within their scaffolds to identify novel chemotypes of *h*DHODH inhibitors. For this purpose, recombinant *h*DHODH was produced using the *E. coli* BL21­(DE3) strain as the expression system. Its construct included an *N*-terminal polyhistidine *tag* to assist the purification by Immobilized Metal Affinity Chromatography (IMAC) using Nickel-NTA (Ni-NTA) resin (Figure S1A), and additional purification was achieved via Size Exclusion Chromatography (SEC) using a Superdex 200 Increase 10/300 GL column (Figure S1B). The inhibitory activity, expressed by half-maximal inhibitory concentration (IC_50_) and the inhibition constant (*K*_i_), was assessed by comparison of the enzyme’s native kinetic parameters [Michaelis–Menten constant (*K*_M_) for the substrate DHO = 32.98 μM; maximal reaction velocity (Vmax) = 3.97 μM/min] (Figure S2) with the ones evaluated in the presence of the compound under investigation. Arzanol emerged from the screen as a one-digit micromolar inhibitor (IC_50_ = 2.02 μM, Figure 3A). Analysis of the *K*_i_ inhibition constant (*K*_i_ = 4.22 μM) showed that arzanol behaves as a noncompetitive inhibitor (Figure 3B).

**Figure 3:**
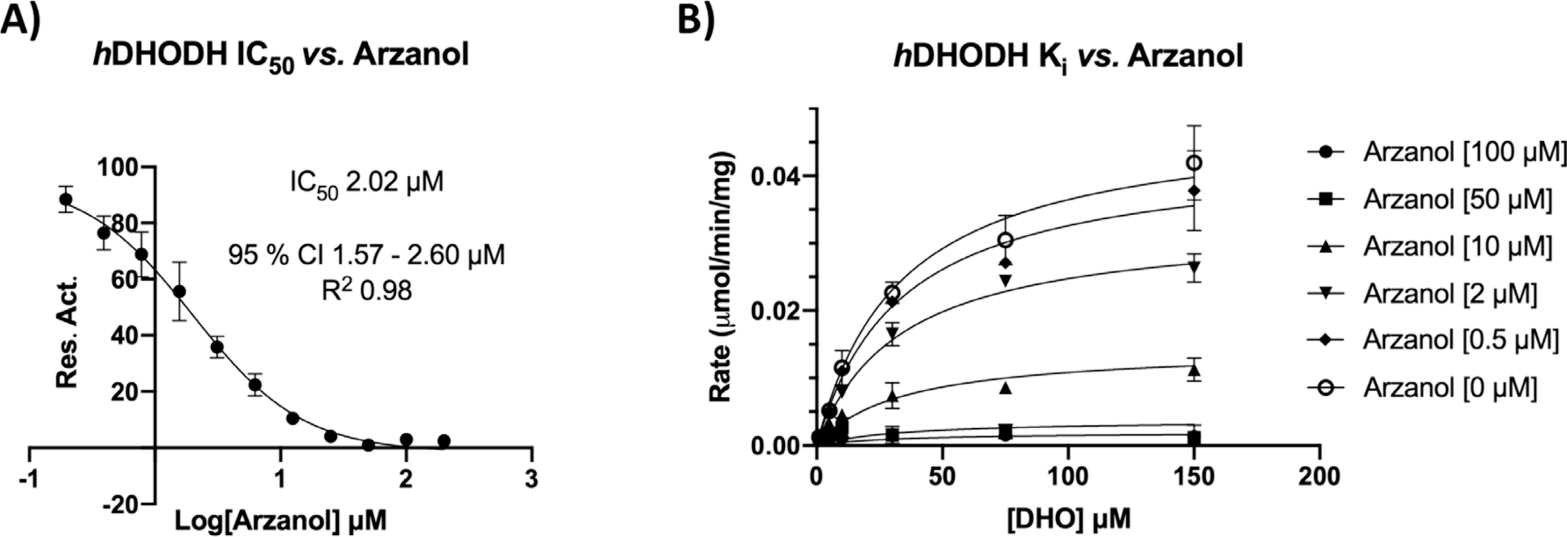
Graphical representation of *h*DHODH inhibition by arzanol. (A) IC_50_ determination with arzanol tested at concentrations ranging from 0.39 to 200 μM, with reactions initiated by the addition of 100 μM DHO as a substrate. (B) *K*_i_ estimation with arzanol tested at concentrations ranging from 0.5 to 100 μM, with reactions initiated by the addition of DHO at varying concentrations (0, 1, 5, 10, 30, 75, and 150 μM). Enzymatic activity was monitored spectrophotometrically at λ = 600 nm. Data are presented as the mean of two independent experiments and were fitted using GraphPad software (95% CI = 95% Confidence Interval).

These encouraging biochemical data provided a rationale to investigate the structural details of the enzyme–inhibitor complex. To this purpose, the purified monomeric enzyme was incubated for 1 h at 4 °C with 2 mM of the product of ORO and 2 mM arzanol prior to cocrystallization. Crystals were obtained using the hanging-drop vapor diffusion method, forming 2 μL drops composed of a 1:1 ratio of the protein-inhibitor-preformed complex to the precipitant solution. Cubic crystals appeared after approximately 2 months; these were then subjected to flash freezing in liquid nitrogen and sent to the European Synchrotron Radiation Facility (ESRF) in Grenoble, France, for X-ray diffraction analysis. The crystal structure of the protein in complex with ORO, the FMN cofactor, and arzanol diffracted at high resolution (1.6 Å), resulting in high-quality electron density (Figure S5A), enabling detailed examination of the binding pocket and the identification of key elements for the modulation of the bioactivity.

In the enzyme–inhibitor complex, arzanol occupies the quinone binding site of *h*DHODH, a feature also common to other inhibitors. Nevertheless, the binding mode of arzanol unveils significant details that were not highlighted in the previously investigated *h*DHODH inhibitors.^16,34,46,47^ Although they are in the same binding site as brequinar, a well-known *h*DHODH inhibitor, several notable differences can be observed when comparing their binding poses within the CoQ10 binding site (Figure 4A,B). The crucial interaction with the essential residue R135 ^48^ is maintained, and it is established by a polar interaction through its α-pyrone hydroxyl group. However, the experimental electron density revealed that when in complex with arzanol R135 adopts two distinct arrangements, enabling the formation of polar contacts with the polyhydroxylated ring in both spatial orientations. Given the intrinsic symmetry of the arzanol molecule, polar contacts could potentially be established by the hydroxyl groups located on both ring A and ring B. However, as discussed above, ring A is distinguished by a vinyl hydroxyl group that is significantly more acidic than the phenolic hydroxyls present on ring B. The vinyl OH on ring A therefore plays a pivotal role in dictating the orientation of the molecule, ensuring that the strongest interaction (an electrostatic interaction with R135) forms preferentially with the most acidic hydroxyl group. Moreover, the ring A is engaged with H-bond interactions with the Y355 and H55 of *subsite 3*, both contributing to the proper orientation of arzanol in the binding pocket.

**Figure 4:**
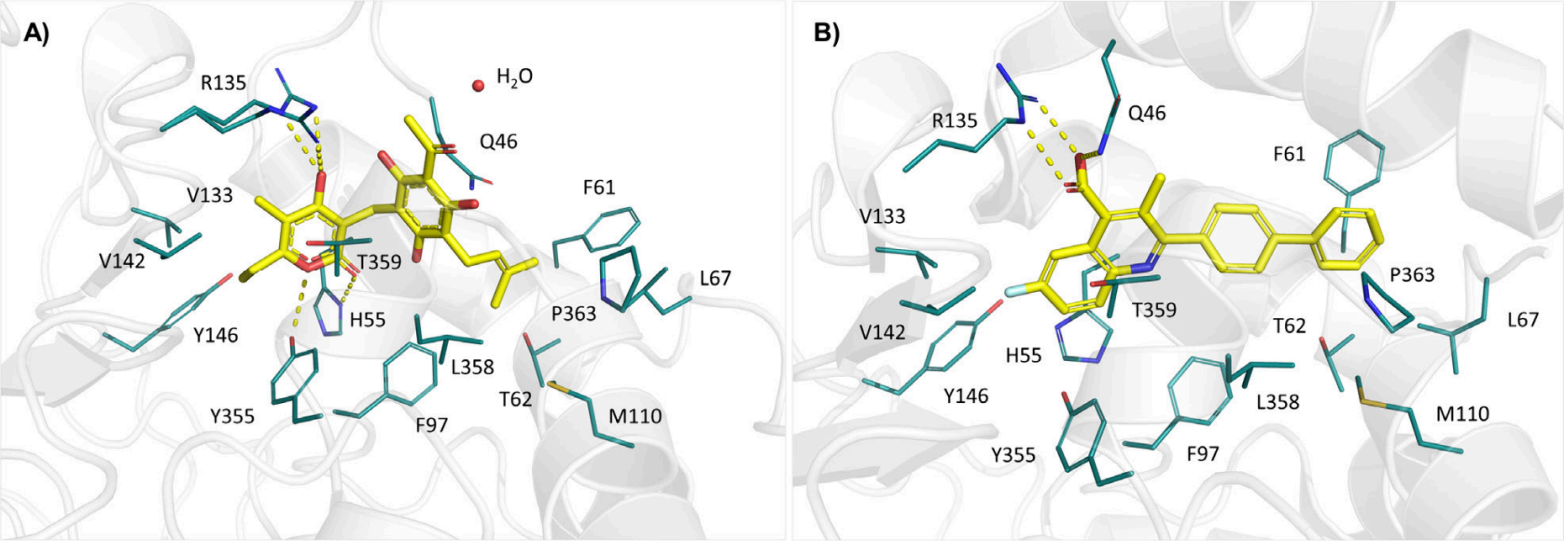
Comparison of the pharmacophoric models of *h*DHODH, highlighting active site residues (cyan sticks), based on X-ray crystallographic structures in complex with (A) arzanol (PDB: 9S1I); and (B) brequinar (PDB: 1D3G), shown in yellow sticks within the CoQ10 binding pocket.

The methylene bridge connecting the aromatic fragment and the pyronic core allows their mobility and the adoption of a “*butterfly shape*” conformation and grants the involvement of the glutamine residue at position 46 of the peptide sequence (Q46). The interaction with this residue could be favored by the planarity of the aromatic ring together with the interaction of the sp^2^ carbons also belonging to the prenyl residue. Remarkably, the side chain of Q46 undergoes a conformational change, assuming an orientation different from the one observed in other reported *h*DHODH inhibitors, allowing its side chain to establish hydrophobic contacts with ring B of arzanol (Figure 4A and Figure S5). However, due to the high flexibility of the N-terminal residues in the protein subjected to X-ray diffraction, the experimental electron density for residues 1–45 was completely undefined. Consequently, atomic coordinates have only been assigned starting from residue Q46, which corresponds to the first visible residue in the X-ray solved structure. This is presumably due to its stabilization by both a water molecule and the bound arzanol compound, which reduced the B-factors. While suggesting a surprising conformational flexibility for Q46, these findings, by disclosing a novel logic of binding, could also inspire the design of novel inhibitors.

To further investigate the dynamic behavior of the crystallographically resolved *h*DHODH–arzanol complex and to complement the static structural view, molecular dynamics (MD) simulations were performed. The protein–ligand complex was immersed in an orthorhombic box of TIP3P water molecules, neutralized, and equilibrated under physiological conditions. Subsequently, a 100 ns MD simulation was conducted at 300 K and 1 bar pressure using the Desmond software within Schrödinger’s Maestro suite.

The stability of the system was monitored by calculating the root-mean-square deviation (RMSD) for all Cα atoms of the protein, as well as for the ligand. The protein maintained remarkable stability, with RMSD values ranging between 1.0 and 1.5 Å throughout the simulation, while the bound ligand exhibited an even lower deviation, consistently below 1.0 Å (Figure 5). These results indicate that the complex remains highly stable under the simulated conditions.

**Figure 5:**
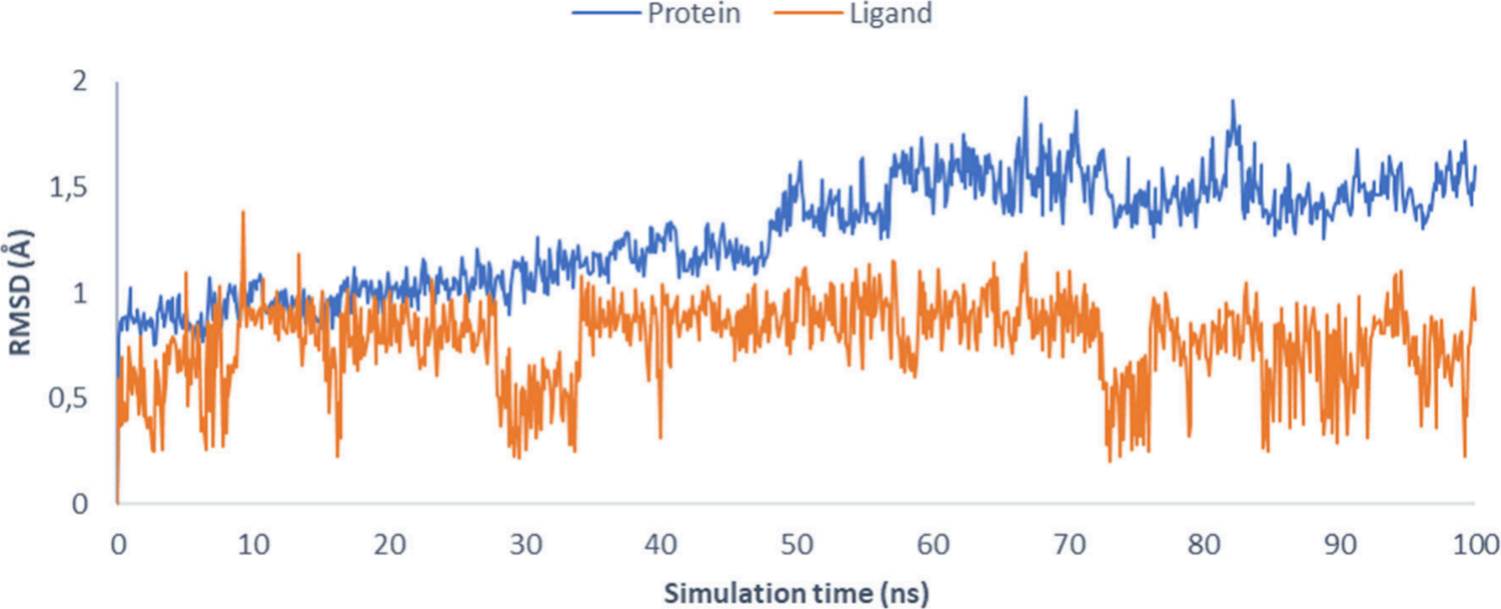
RMSD with respect to the initial structures as functions of simulation time (ns). In blue, the RMSD for Cα atoms (Å) of the protein, and in orange, the RMSD for heavy atoms of the ligand.

Further analysis of the trajectories confirmed that the key interactions observed in the crystallographic structure of the *h*DHODH–arzanol complex were preserved during the entire simulation period (Figure 6). This finding highlights the robustness of the binding mode and supports the physiological relevance of the crystallographically determined pose.

**Figure 6:**
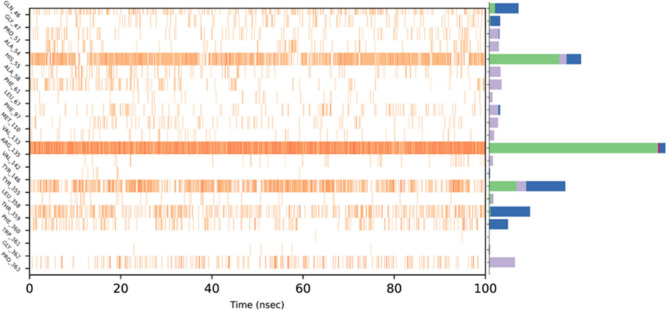
Timeline representation of the interactions and protein–ligand contacts. The left side shows which residues interact with the ligand in each trajectory frame. Some residues make more than one specific contact with the ligand, which is represented by a darker shade of orange. The right side shows the interactions categorized by type: Hydrogen Bonds (green), Hydrophobic Bonds (violet), Ionic Bonds (purple), and Water Bridges (blue).

In addition, the binding free energy of the complex was estimated by using the MM-GBSA technique. The resulting interaction energy of −64.02 kcal/mol further corroborates the stability of the complex and provides quantitative support for the favorable binding of arzanol to *h*DHODH (Table 2).

**Table 2:** MM-GBSA Energies for the Docking Pose of Ligand Binding at the Active Site

	Δ*G* Binding	Coulomb	Covalent	H-bond	Lipo	Packing	Solv_GB	vdW
ARZ	–64.02	–157.92	2.29	–1.73	–19.15	–1.42	173.59	–59.68

To validate the translation of enzyme inhibition into bioactivity, arzanol was then assayed for antiviral activity against the SARS-CoV-2 Italian strain PV10734 (D614G, lineage B.1.1) using an *in vitro* model based on cytopathic effect (CPE) inhibition.^49^ Arzanol showed only marginal cytotoxicity [CC_50_ = 160.1 ± 2.2 μM], and noncytotoxic concentrations (≤75 μM) were used for the antiviral assays (Figure S3). The CPE-inhibition assay^50^ was adapted to include four treatment conditions, corresponding to distinct times of arzanol administration (PRE- or POST-infection) and, after a 4-h exposure, to the presence (PRE+ and POST+) or absence (PRE– and POST−) of readdition during the subsequent 72 h of incubation (see the Experimental Section for details). Microscopic observation of cells at 72 h postinfection (hpi) revealed that arzanol reduced SARS-CoV-2-induced CPE in a concentration-dependent manner. The most marked inhibition of CPE was observed under PRE+ and POST+ conditions, where arzanol was readministered after the initial 4 h exposure, suggesting that prolonged presence of the compound enhances its antiviral activity. In contrast, PRE– and POST– treatments showed a milder protective effect with less pronounced inhibition of CPE, which was observed only at the 75 μM concentration. The extent of viral replication was quantified by end point titration and expressed as Tissue Culture Infectious Dose per mL (TCID_50_/mL).^51,52^ Arzanol significantly reduced viral titers of progeny viruses in the PRE+ and POST+ conditions, with the strongest reduction observed at concentrations of 75 and 37.5 μM (Figures 7and 8). Conversely, only a modest reduction was observed in the POST– condition at 75 μM, while no significant reduction was noted in the PRE– condition. The IC_50_ values were 18.8 μM (PRE+) and 15 μM (POST+), corresponding to Selectivity Index (SI) values of 8.5 and 10.7, respectively, indicating a favorable balance between antiviral potency and cytotoxicity (SI = CC_50_/IC_50_). These results support a correlation between continuous compound exposure and enhanced viral suppression. Overall, in agreement with the biochemical IC_50_ on the isolated enzyme, these findings underscore the good antiviral activity of arzanol against SARS-CoV-2 *in vitro*, with enhanced efficacy when maintained throughout the infection period.

**Figure 7:**
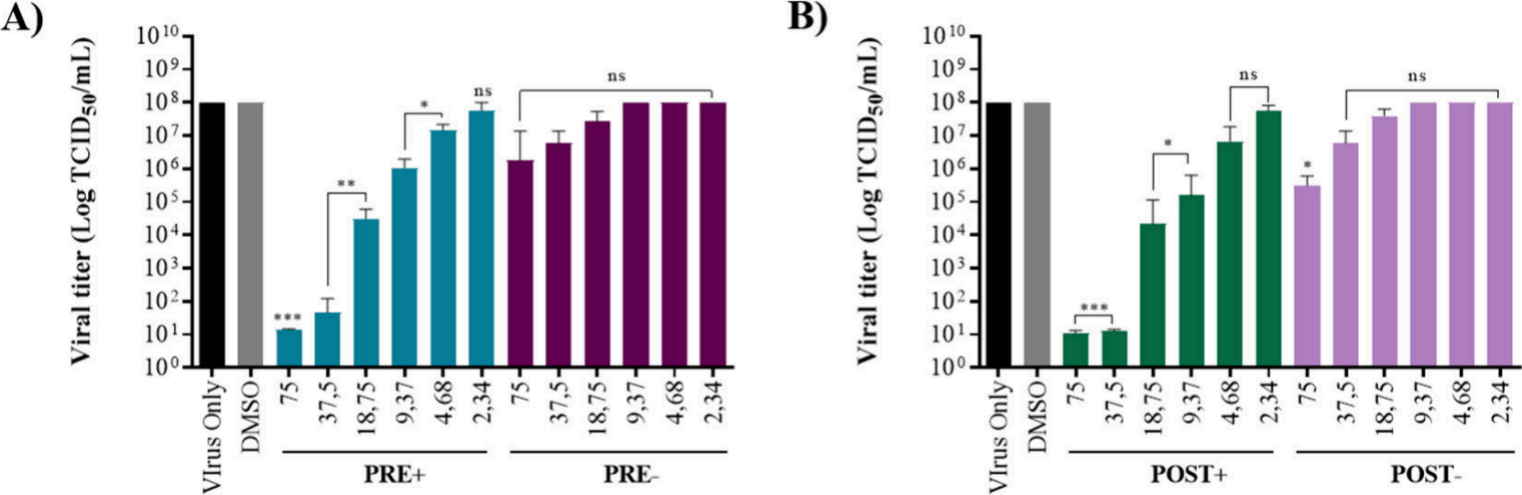
Effect of arzanol on viral titers of SARS-CoV-2 Italian strain PV10734 by the TCID_50_/mL assay. The virus titers (log TCID_50_/mL) in the supernatants of Vero E6 cells infected with SARS-CoV-2 PV10734 and treated with arzanol were determined by the Tissue Culture Infectious Dose per mL (TCID_50_/mL) assay, calculated using the Reed and Muench method. Data were collected from four treatment conditions: (A) PRE+ (preinfection with readdition) and PRE– (preinfection without readdition) and (B) POST+ (postinfection with readdition) and POST– (postinfection without readdition). Data are presented as the mean ± SD from three independent experiments (***p ≤ 0.001; **p ≤ 0.01; *p ≤ 0.05; ns, *p* ≥ 0.05) and were fitted by using GraphPad software.

**Figure 8:**
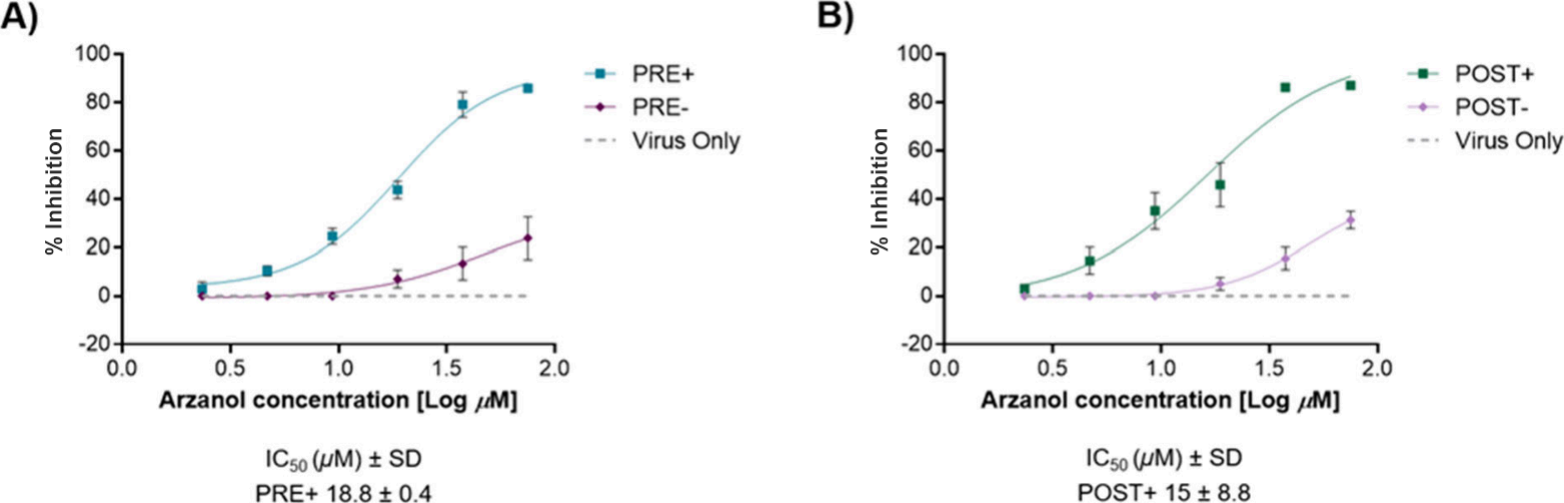
% Inhibition of SARS-CoV-2 Italian strain PV10734 and the IC_50_ of arzanol. (A) PRE+ (preinfection with readdition) and PRE– (preinfection without readdition); (B) POST+ (postinfection with readdition) and POST– (postinfection without readdition). The *y*-axis indicates the inhibition of virus titer (percent) relative to that of the untreated control group (Virus Only). The *x*-axis indicates the concentration (log) of arzanol. Data were fitted by using GraphPad software.

## Conclusions

Arzanol has been reported to exhibit a distinct range of biological activities, including, in addition to antimicrobial activity, actions as anti-inflammatory and antiviral, which could be recapped by the inhibition of *h*DHODH. Although less potent than some synthetic inhibitors, such as brequinar (IC_50_ of 4.5 nM), arzanol is nevertheless interesting as a *h*DHODH modulator (IC_50_ 2.02 μM) because of its unique docking to the allosteric site of the enzyme, a finding which could inspire the *de novo* design of inhibitors as well as assist the optimization of the activity of the natural product. Our structural insights unveil both canonical and noncanonical interactions within the enzyme’s active site: while arzanol does not fully exploit the extensive network of contacts that stabilize high-affinity inhibitors in the subnanomolar range, it induces a distinct rearrangement of several residues in the CoQ10 binding pocket. This rearrangement, including unconventional displacements of residues Q46 and R135, not only underscores the conformational plasticity of *h*DHODH but also suggests innovative strategies for structure-guided drug design. Specifically, our findings indicate that decorating the arzanol scaffold to recover interactions with N-terminal residues that are partially disengaged in the current binding mode could substantially improve potency. In this regard, while ring A seems to be a good source of effective binding with *subsites 2* and *4*, the polarity present in ring B probably is not optimal in engaging effective binding with the lipophilic residues characterizing *subsite 1*, resulting in reduced IC_50_ at the enzymatic level. However, because there is space for optimizing ring B, the next step will be directed to strategically modulate this substructure in order to promote hydrophobic interactions within the LP, providing a promising basis for future synthetic or semisynthetic derivatization approaches.

Our enzymatic data were validated in assays of antiviral activity, showing that arzanol can reduce SARS-CoV-2-induced cytopathic effects and viral titers. The favorable micromolar potency of arzanol provides an excellent foundation for medicinal chemistry efforts aimed at restoring significant interactions at the N-terminal domain of *h*DHODH, while it exploits the plasticity of adjacent regions. Such optimization strategies have the potential to deliver novel therapeutics that address not only viral infections but also cancer and autoimmune diseases, where *h*DHODH plays a pathogenic role, further broadening the potential impact of optimized arzanol derivatives.

Arzanol has been reported to engage with distinct macromolecular targets (NF-κB, mPGES-1, bGP, Sirt1),^37,53–55^ a surprising observation for a compound devoid of covalent reactivity. This polypharmacological profile raises the possibility of limited selectivity, raising concerns about off-target interactions or multiple side effects. While this feature represents a potential limitation, it is also not uncommon among natural products and may even provide therapeutic advantages in complex diseases, such as inflammation or cancer, where simultaneous modulation of multiple pathways can be beneficial.

In this study, we present the first crystal structure of a target in complex with arzanol, paving the way for rational optimization aimed at improving both potency and selectivity. In principle, the same approach could be extended to other reported targets of arzanol, through experimental or *in silico* structure-based analyses, to guide the synthesis of derivatives with refined selectivity profiles. Arzanol’s binding promiscuity may stem from its tautomeric variability and conformational flexibility around the methylene pivot, a structural “ballet” often exploited by natural products to expand the inventory of their molecular targets.^56^

Although this promiscuity may currently limit arzanol’s direct therapeutic use, the biochemical and structural insights reported here lay the groundwork for future studies, including target profiling, SAR exploration, and toxicity evaluation, necessary to clarify its therapeutic potential and safety window.

## Experimental Section

### General Experimental Procedures

Arzanol was isolated by employing silica gel 60 (0.063–0.200 mm), reversed-phase (RP) C18 silica gel (25 μm), and Celite 545 (particle size 0.02–0.1 mm) used for low-pressure liquid chromatography (LPC) and vacuum chromatography; the reagents were purchased from Macherey-Nagel (Düren, Germany). Purifications were monitored by TLC on Merck 60 F254 (0.25 mm) plates and visualized by staining with 5% H_2_SO_4_ in EtOH and heating. Solvents were obtained from Sigma-Aldrich (Milan, Italy). ^1^H NMR 400 MHz spectra were acquired on Bruker 400 spectrometers (Bruker, Billerica, MA, USA). Chemical shifts were referenced to the residual solvent signal (C_3_D_6_O δ_H_ 2.09). The powder was dissolved in DMSO immediately before use and stored at −20 °C. The open reading frame encoding *h*DHODH was cloned into a pET19 plasmid (service provided by GenScript). The His-tagged *h*DHODH was expressed in the *E. coli* BL21 (DE3) bacterial strain, purified using Ni-NTA and Superdex 200 resins, concentrated with Amicon Ultra filters, and assessed for purity by SDS-PAGE analysis. All enzymatic assays were performed in triplicate in 96-well plates at 37 °C using a TECAN Sunrise spectrophotometer. Protein concentrations were determined by the Bradford assay. GraphPad Prism (version 8.4.2) was used for nonlinear regression analyses, kinetic parameter estimation, and IC_50_/*K*_i_ calculations. Cell culture experiments were performed using standard conditions (DMEM, 10% FBS, 2 mM l-glutamine) at 37 °C with 5% CO_2_. All SARS-Cov-2 infection experiments were carried out in a BSL-3 facility, following approved safety protocols. All of the reagents and solvents were purchased from commercial suppliers and used without further purification.

### Isolation of Arzanol from Aerial Parts of *H. microphyllum* (Willd.) Cambess. subsp. *thyrrenicum* Bacch. Brullo and Giusso

#### Plant Material

Flowered aerial parts of *H. microphyllum* (Willd.) Cambess. subsp. *thyrrenicum* Bacch. Brullo and Giusso were collected around Monteponi (Sardinia) at the beginning of July 2019. The plant material was identified by Prof. Cinzia Sanna, and a voucher specimen (He_2019) is kept at the Phytochemistry laboratory in Novara.

#### Arzanol Isolation

500 g of flowered aerial parts of *H. microphyllum* was powdered and extracted with acetone (ratio acetone/plant material 10:1 vol/weight) in a 10 L percolator at RT (2 extractions of 12 h). After the extraction, the solvent was filtered to remove the vegetal material, and the resulting acetonic fraction was evaporated at reduced pressure to obtain a dried extract of 21 g as brown gum, which was dissolved in a minimal amount of acetone; then, silica gel was added (1:3 weight/weight g) to obtain a suspension that was completely evaporated under reduced pressure. The powder obtained in this way was stratified on a layer of Celite (1:3 weight/weight g) moistened with petroleum ether and protected on its surface by filter paper in a sintered funnel with a side arm for vacuum connection. Next, solvents of increasing polarity were subsequently added: petroleum ether (PE), ethyl acetate (EtOAc), and tetrahydrofuran (THF), in 1:30 wt/volume mL, and they were sequentially passed through the filter. The three vacuum filtrates (PE, EtOAc, and THF) were collected separately and evaporated. Each fraction was then analyzed with ^1^H NMR (Figure S4) to detect the presence of the phloroglucinol arzanol that has been revealed in the 10 g EtOAc fraction. This latter fraction was fractionated by chromatography on silica gel (250 g, PE-EtOAc gradient from 70:30 to 40:60) to afford 310 mg of arzanol (0.062%) as a yellow powder after diethyl ether crystallization. The compound was identified by ^1^H NMR according to the literature.^36,57^

### Protein Expression and Purification

*h*DHODH was expressed and purified according to a previously described procedure.^46^ Both the purified protein and the crystals were yellow because of the presence of the FMN cofactor in the protein core. FMN was not exogenously added during the protein production steps but was directly integrated into the enzyme during its recombinant expression. In fact, the heterologous protein extracts the key FMN cofactor from the expression system (*E. coli*).

### Enzymatic Inhibition Assays

The activity of *h*DHODH was monitored by measuring the reduction of the oxidized form of 2,6-dichloroindophenol (DCIP) at λ = 600 nm, with each DHO oxidation cycle generating colorless DCIPH_2_ in a coupled reaction.

#### IC_50_ Evaluation

The purified recombinant protein was tested at 100 nM alone and in complex with arzanol (tested at increasing concentration from 200 to 0.195 μM IC_50_ in 1:2 ratio serial dilution). After 20 min of preincubation at 37 °C in a mixture of 50 mM Tris-HCl, pH 8, 400 mM NaCl, 5% (volume/volume) glycerol, 1 mM EDTA, 0.1% (volume/volume) Triton X-100, 0.1 mM CoQ10 (dissolved in DMSO from a 5 mM stock solution), and 0.05 mM DCIP up to a final volume of 100 μL, the reaction was initiated with the addition of DHO at 100 μM. The reduction of DCIP was monitored at 600 nm for 10 min. The curves were analyzed for their maximum drop in the first 5 min of the enzymatic reaction, and the enzymatic activity was normalized against *h*DHODH activity in the absence of an inhibitor. The IC_50_ value was calculated with GraphPad Prism (version 8.4.2) software as the mean of three independent experiments.

#### Michaelis–Menten Kinetics

The assay was performed as described above, but the reaction was initiated by the addition of the substrate DHO at increasing concentrations from 0.49 to 500 μM in a 1:2 ratio serial dilution. For the calculation of the *K*_M_ parameter, curves were analyzed for their maximum drop in the first 3 min of the enzymatic reaction. Biochemical parameters (Vmax and *K*_M_) were calculated using GraphPad Prism (ver. 8.4.2) as the mean of three independent experiments.

#### Inhibition Studies for the Calculation of *K*_i_

In a 96 well-plate, six fixed concentrations of arzanol (from 0 to 100 μM) were tested toward 100 nM *h*DHODH by varying DHO concentrations from 1 to 150 μM. The reaction started with the addition of the substrate DHO, and the reduction of DCIP was monitored through a TECAN Sunrise spectrophotometer at 600 nm for 5 min. The resulting curves were analyzed for their maximum drop, and the *K*_i_ biokinetic parameter was calculated using GraphPad Prism software (version 8.4.2) as the mean of three independent experiments.

### Co-crystallization Experiments

The monomeric purified protein was concentrated up to 15 mg/mL and preincubated overnight at 4 °C with final concentrations of 2 mM DHO and 2 mM arzanol. The protein–inhibitor complex (2 μL) was mixed with 2 μL of reservoir solution consisting of 2 M ammonium sulfate, 100 mM sodium acetate, pH 4.8, and 20% volume/volume glycerol in a hanging drop crystallization assay. After 3 months, yellow cubic crystals of the protein in complex with arzanol were obtained.

### X-ray Data Collection, Structure Determination, and Refinement

Protein crystals were flash-cooled in liquid nitrogen and sent to the European Synchrotron Radiation Facility (ESRF), France, where they underwent an X-ray diffraction experiment on the beamline ID30-B^58^ using an Eiger2_9 M as a detector. The collected data were indexed, integrated, and scaled to a resolution of 1.4 Å using the Aimless utilities of the CCP4i2 Program Suite version 8.0.008.^59^ The structure was determined by molecular replacement with Phenix-PHASE^60^ using the structure of *h*DHODH from 7Z6C PDB as a search model. Manual model building was performed with the Coot program,^61^ and pictures were generated with PyMol.^62^ Data collection and refinement statistics are listed in Table S1.

### PDB Deposition

The atomic coordinates and structure factors of *h*DHODH in complex with arzanol have been deposited in the Protein Data Bank (PDB) as the 9S1I code.

### Molecular Dynamics Simulations

A molecular dynamics protocol was used to analyze the X-ray structure. The protocol employed was executed using the Desmond module within the Schrödinger Small-Molecule Drug Discovery Suite (Small-Molecule Drug Discovery Suite 2025-1, Schrödinger, LLC, New York, NY, 2025). The simulation system was prepared to mimic physiological conditions, employing explicit solvent modeling with the TIP3P water model and an orthorhombic box measuring 10 × 10 × 10 Å, supplemented with 0.15 M salt. To ensure electrical neutrality of the entire simulation system, counterions, specifically Na^+^ and Cl^–^, were added at a minimum distance of 20 Å from the ligand within the simulation system. Subsequently, the system underwent relaxation in multiple stages: (i) initial relaxation involved up to 2000 minimization steps with a force constant of 50 kcal/mol/Å^2^, applying harmonic restraints to the solute atoms; (ii) a 12 ps MD simulation was conducted at 10 K with a force constant of 50 kcal/mol/Å^2^. This was carried out under the NPT ensemble, Berendsen thermostat, and barostat, all while retaining harmonic restraints. (iii) The system was then heated from 10 to 300 K over a 24 ps period with harmonic restraints still in place. The NPT ensemble, Berendsen thermostat, and barostat were used for this phase; (iv) a 24 ps MD simulation at 300 K without harmonic restraints was performed using the NPT ensemble, Nose-Hoover thermostat, and Martyna–Tobias–Klein barostat. Following the system’s relaxation, each docked complex underwent a 100 ns MD simulation using default parameters under the NPT ensemble. Upon completion of the MD simulations, the resulting trajectory was analyzed, and key parameters, including the root-mean-square deviation (RMSD) and protein–ligand contacts, were computed using simulation interaction diagrams.

The ligand–residue free energies of binding calculations were performed by the molecular mechanics/generalized born surface area (MM/GBSA) method available in the Schrödinger Suite. The equation used to calculate the binding energy is as follows:

$$\Delta G_{\mathrm{bind}}=\Delta E_{\mathrm{MM}}+\Delta G_{\mathrm{solv}}+\Delta G_{\mathrm{SA}}$$

where Δ*E*_MM_ is the difference in minimized energies as follows:

$$\Delta E_{\mathrm{MM}}=E_{\left(\mathrm{complex}\right)}-E_{\left(\mathrm{ligand}\right)}-E_{\left(\mathrm{receptor}\right)}$$

The difference in the GBSA solvation energy of the complex and the sum of ligand and protein solvation energies is denoted by Δ*G*_solv_. Also, Δ*G*_SA_ is the difference in the surface area energy of the complex and the sum of the protein and ligand. A script was used to calculate the average MM-GBSA binding energy, which also generates Coulomb energy (Coulomb), covalent binding energy (Covalent), Hydrogen-bonding energy (H-bond), lipophilic energy (Lipo), Generalized Born electrostatic solvation energy (Solv_GB), and van der Waals energy (vdW).

### Cell Culture

Vero E6 cells (ATCC CRL-1586) were maintained in Dulbecco’s Modified Eagle’s Medium (DMEM; EuroClone, Milan, Italy) supplemented with 10% fetal bovine serum (FBS; Gibco, Thermo Fisher, Monza, Italy), 2 mM l-glutamine (EuroClone, Milan, Italy), and 1% penicillin–streptomycin (EuroClone, Milan, Italy). Cells were routinely subcultured twice weekly at a 1:4 ratio using a trypsin-EDTA solution (EuroClone, Milan, Italy).

### Viral Strain and Titration

The clinical isolate of SARS-CoV-2 Italian strain PV10734 (D614G, lineage B.1.1) was obtained from Fondazione IRCCS Policlinico San Matteo (Pavia, Italy), where they were propagated in Vero E6 cells as previously described^63,64^ and stored at −80 °C. The viral titer was determined by the TCID_50_/mL method.^65^ All infection experiments were carried out in a biosafety level 3 (BLS-3) laboratory.

### Cytotoxicity Assays

Activity was assessed in Vero E6 cells seeded in 96-well plates at a density of 2 × 10^4^ cells/well and incubated overnight at 37  °C in a humidified atmosphere containing 5% CO_2_. Arzanol was dissolved in DMSO and serially diluted to final concentrations of 2.3, 4.7, 9.4, 18.8, 37.5, 75, 150, and 300  μM. The final DMSO concentration was adjusted to 0.5% (v/v) in all wells. After 72 h of treatment, cytotoxicity was quantified using the Cytotoxicity LDH assay kit–WST (Dojindo Molecular Technologies Inc.), following the manufacturer’s instructions. Absorbance was measured at 490 nm, and the percentage cytotoxicity was calculated relative to the DMSO control. The 50% cytotoxic concentration (CC_50_) was determined by a nonlinear regression dose–response curve using GraphPad Prism (version 8.4.2).

### SARS-CoV-2 Cytopathic Effect (CPE) Assay

The described assay^51,52^ was modified as follows: Vero E6 cells were seeded in 96-well plates (2 × 10^4^ cells/well) and incubated overnight at 37 °C in a humidified 5% CO_2_ atmosphere. The SARS-CoV-2 clinical isolate PV10734 (D614G, lineage B.1.1) was titrated and used at a final concentration of 100 TCID_50_/well. Arzanol was dissolved in DMSO and serially diluted from 75 to 2.3 μM in DMEM supplemented with 2% FBS. DMSO was used as the vehicle control. The experimental design included two main treatment conditions—preinfection and postinfection, each subdivided based on whether arzanol was readministered after the initial treatment phase. In the preinfection condition, cells were first exposed to different concentrations of arzanol for 4 h. After incubation, arzanol was removed by washing with PBS, and the virus was added in 2% FBS-DMEM for 2 h to allow adsorption. Cells were then washed again and incubated either with fresh complete medium containing arzanol at the original concentrations (PRE+) or with the medium alone (PRE−). In the postinfection condition, cells were first infected with the virus for 2 h. After viral adsorption, cells were washed and treated with arzanol for 4 h at different concentrations. Following this treatment, cells were washed again and incubated either with fresh medium containing arzanol (POST+) or with medium alone (POST−). All conditions were then incubated for 72 h at 37 °C in 5% CO_2_. CPE in each well was observed by microscopy at 72 hpi, and supernatants were collected in 1.5 mL microfuge tubes and stored at −80 °C. Arzanol activity was compared with untreated, uninfected cells (cell control, 100% activity) and untreated infected cells (virus only, 0% activity).

### IC_50_ Determination by Tissue Culture Infectious Dose (TCID) Assay

Vero E6 cells were seeded in quadruplicate in 96-well plates at a density of 2 × 10^4^ cells/well in complete DMEM and incubated overnight at 37 °C in a 5% CO_2_ atmosphere. The following day, the supernatant from each CPE assay condition involving arzanol was subjected to serial 1:10 (v/v) dilutions in DMEM containing 2% FBS. These dilutions were added to subconfluent Vero E6 monolayers after removing the culture medium. Plates were incubated for 72 h, and viral titers were determined by the TCID_50_/mL method, calculated according to Reed and Muench.^51,52^ The titers were compared with control conditions, including untreated virus-infected cells (virus only) and virus-infected cells treated with a vehicle (DMSO). Data from three independent experiments were used to calculate the IC_50_ through a nonlinear regression curve by using GraphPad Prism (version 8.4.2).

## Supplementary Material



## Notes

Detailed experimental procedure for arzanol isolation; SDS-PAGE analysis and chromatographic data demonstrating protein purification; cytotoxicity assay protocols and results; ^1^H NMR spectrum for structural validation of arzanol; analysis of arzanol’s chemical interactions within its binding site; table summarizing X-ray crystallography refinement statistics (PDF)
