# Supplementary material for: Arzanol Inhibits Human Dihydroorotate Dehydrogenase and Shows Antiviral Activity
Source: J Nat Prod. 2025 Oct 27;88(11):2586–95. doi: 10.1021/acs.jnatprod.5c00887 (PMC12670699; doi:10.1021/acs.jnatprod.5c00887)
Supplement: Supplementary file 1 [file np5c00887_si_001.pdf]

Supporting Information

Arzanol inhibits human dihydroorotate dehydrogenase and shows anti-viral activity

Marta Alberti[†], *Martina Tamburello*[^], *Stefano Salamone*[†], *Giorgio Gallinella*[§], *Cinzia Sanna*[¶], *Giovanni Battista Appendino*[†], *Marco L. Lolli*[#], *Alberto Massarotti*[†], *Federica Pollastro*[†*], and *Riccardo Miggiano*[†*]

[†] Department of Pharmaceutical Sciences, University of Piemonte Orientale, Via G. Bovio 6, 28100 Novara, Italy;

^ Department of Medical and Surgical Sciences, Alma Mater Studiorum University of Bologna, Via Massarenti 9, 40138 Bologna, Italy;

[§] Department of Pharmacy and Biotechnology, Alma Mater Studiorum University of Bologna, Via Massarenti 9, 40138 Bologna, Italy;

[¶] Department of Life and Environmental Sciences, University of Cagliari, Via Sant'Ignazio da Laconi 13, 09123 Cagliari, Italy;

[#] Department of Sciences and Drug Technology, University of Torino, Via P. Giuria 9, 10125 Torino, Italy;

*Correspondence to: riccardo.miggiano@uniupo.it; federica.pollastro@uniupo.it

Table of Contents:

Figure S1. SDS-PAGE analysis of recombinant *h*DHODH following purification steps. S2
Figure S2. Biochemical characterization of the purified recombinant *h*DHODH. S3
Figure S3. Cytotoxicity of arzanol (CC_{50}) toward Vero E6 Cells. S4
Figure S4. ^{1}H NMR (400 MHz) of arzanol in C_3D_6O. S5
Figure S5: Binding mode of arzanol within the CoQ10 pocket of *h*DHODH. S6
Table S1. Data collection and refinement statistics for X-ray crystallography studies. S7

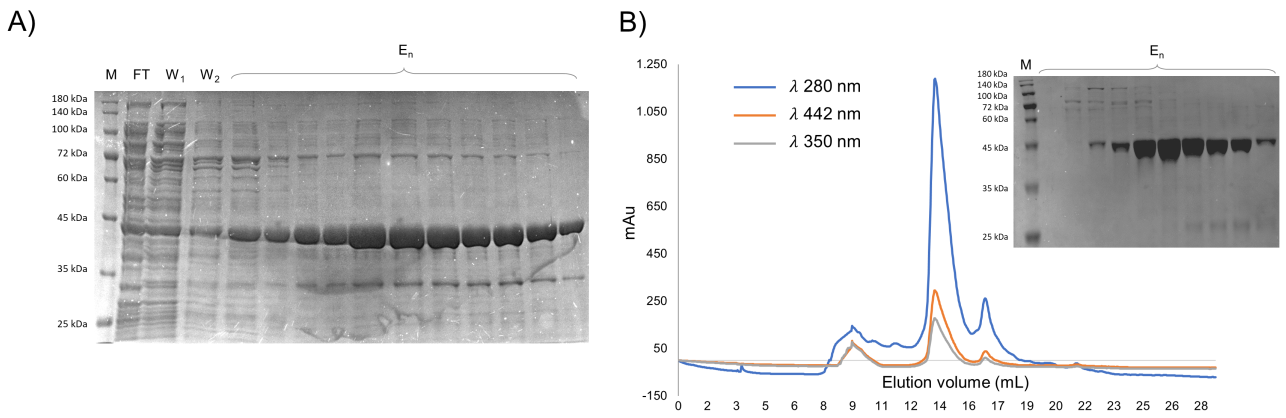

Figure S1. SDS-PAGE analysis of recombinant *h*DHODH following purification steps.

(A) Elution profile of *h*DHODH (MW: 42.57 kDa) after IMAC with Ni-NTA resin - M: molecular weight protein marker; FT: Flow-Through (unbound fraction); $W_{1,2}$: sequential wash fractions with 80 mM imidazole buffer; E_n: progressive eluted fractions in 300 mM imidazole buffer. **(B)** Elution profile of *h*DHODH after SEC with Superdex 200 Increase 10/300 GL column. M: molecular weight protein marker; E_n: progressive eluted fractions in SEC buffer.

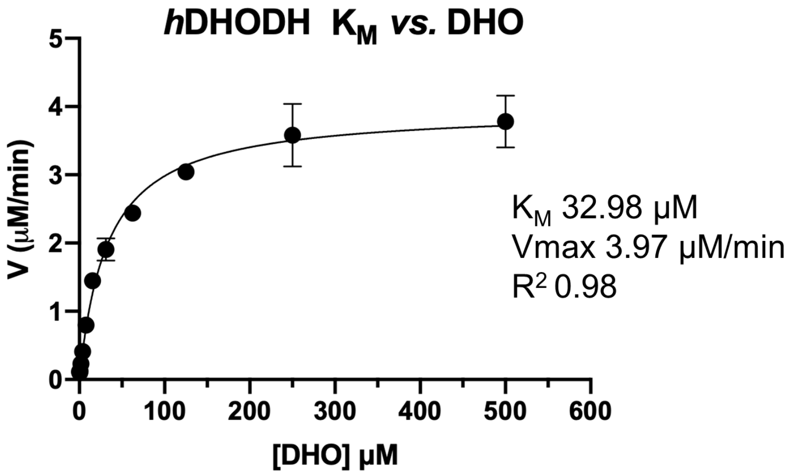

Figure S2. Biochemical characterization of the purified recombinant *h*DHODH.

Michaelis–Menten kinetics of the reaction catalyzed by *h*DHODH in the presence of DHO substrate (0.48, 0.98, 1.95, 3.91, 7.81, 15.63, 31.25, 62.5, 125, 250, and 500 µM). The Michaelis-Menten kinetic model was used to determine the K_M and Vmax values by spectrophotometric assay at λ = 600 nm. Data are presented as the mean of two independent experiments (GraphPad).

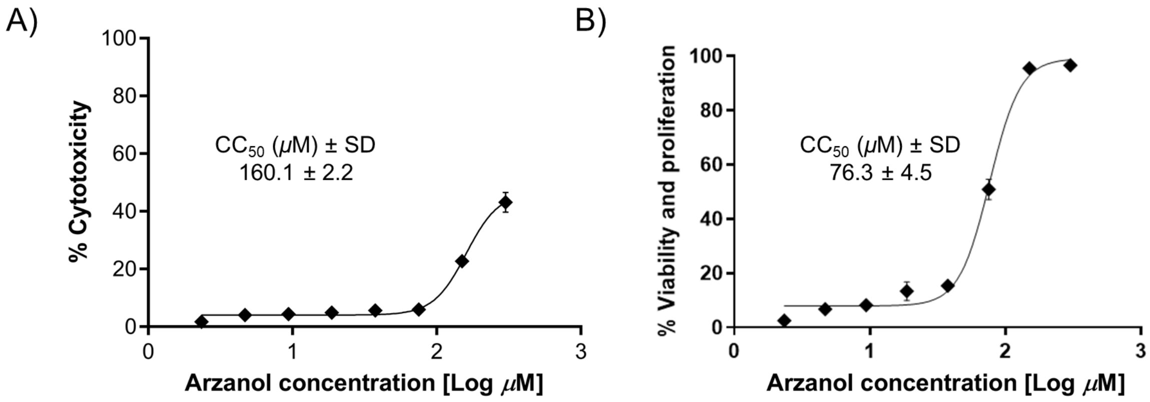

Figure S3. Cytotoxicity of arzanol (CC_{50}) toward Vero E6 Cells.

(A) Vero E6 cells were treated with arzanol at concentrations of 2.3; 4.7; 9.4; 18.8; 37.5; 75; 150 and 300 µM for 72 hours. Cytotoxicity was assessed using **(A)** the LDH Cytotoxicity Assay Kit – WST, where absorbance was measured at 490 nm; **(B)** the Neutral Red Uptake (NRU) assay, where absorbance was measured at 540 nm. The percentage of cell cytotoxicity and cell viability were calculated relative to the DMSO control. Data are expressed as the mean ± SD from three independent experiments (GraphPad).

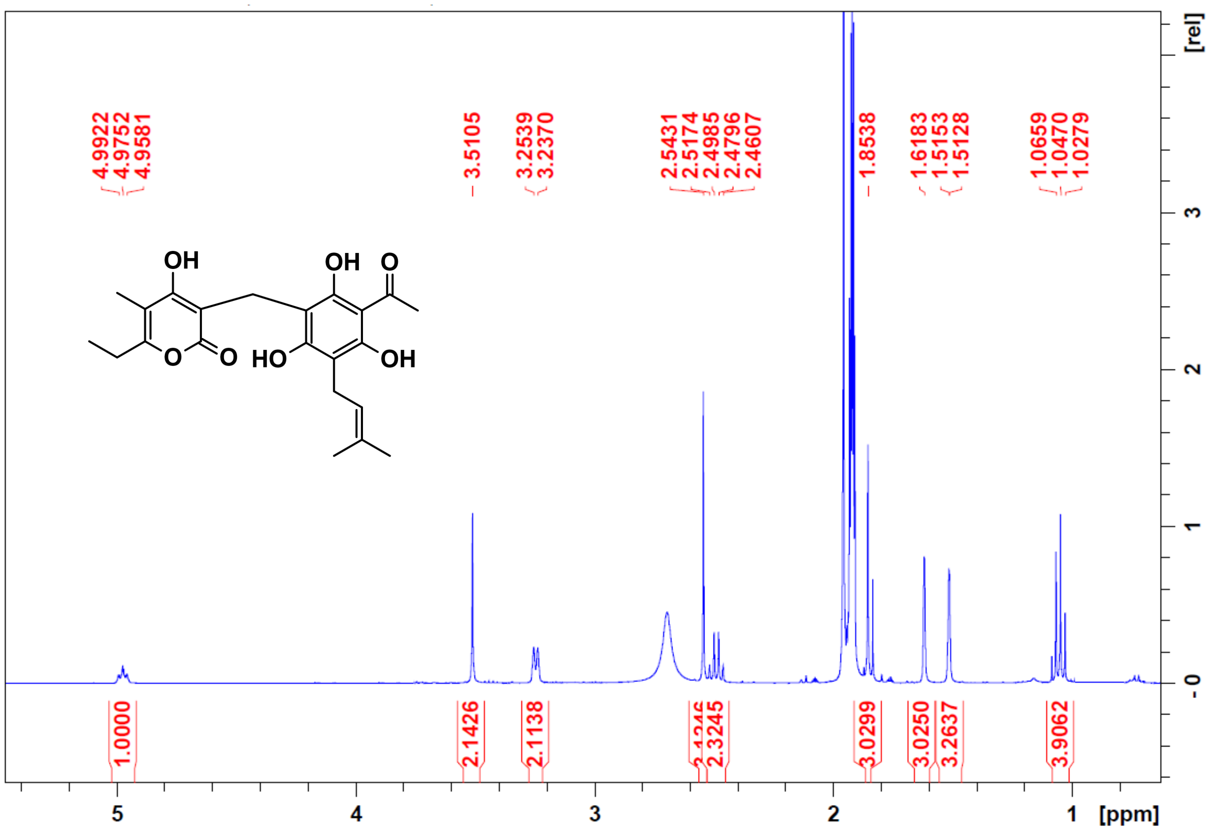

Figure S4. ^{1}H NMR (400 MHz) spectrum of arzanol in C_3D_6O.

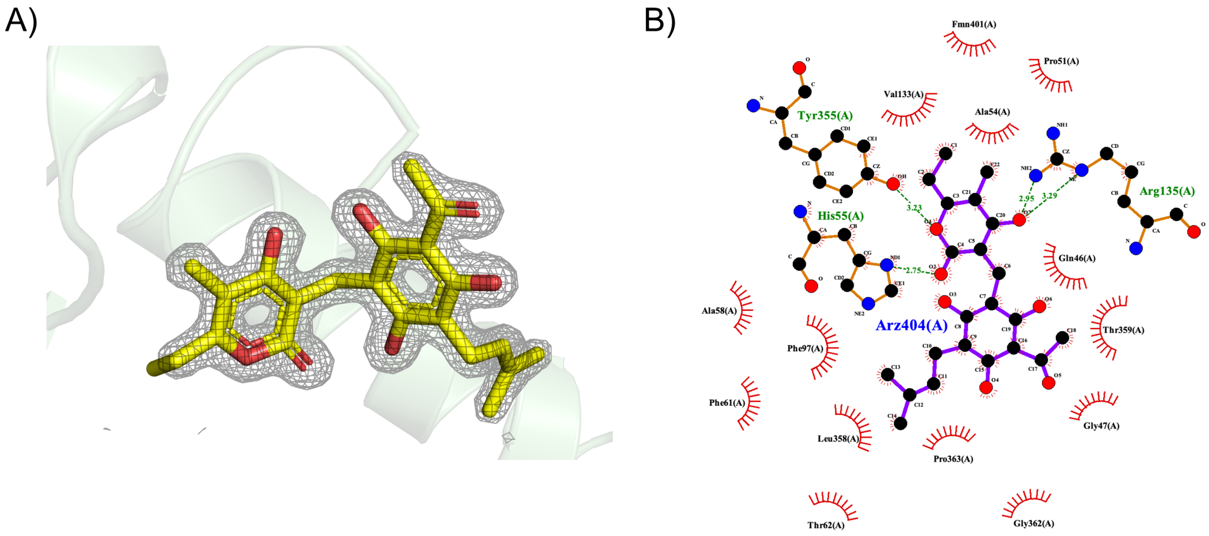

Figure S5: Binding mode of arzanol within the CoQ10 pocket of *h*DHODH.

A) Experimental electron density map (2Fo-Fc, contoured at 1.0 σ, carve 1.6 Å) surrounding arzanol (shown as yellow stick) co-crystallized with *h*DHODH (shown as palegreen cartoon). **B)** LigPlot$^+$ analysis highlighting polar and hydrophobic contacts between arzanol and key residues in the *h*DHODH binding site.

Table S1. Data collection and refinement statistics for X-ray crystallography studies.

Resolution range	48.34 – 1.70 (1.61 – 1.61)
Space group	P 32 2 1
Unit cell	90.48 90.48 122.86 90 90 120
Total reflections	1296740 (192599)
Unique reflections	75934 (10956)
Multiplicity	17.1
Completeness (%)	100 (100)
Mean I/sigma(I)	19.5 (4.4)
R-merge	0.093 (0.605)
R-meas	0.096 (0.624)
R-pim	0.023 (0.148)
CC1/2	0.999 (0.939)
Wilson B factor	16.12
Reflections used in refinement	75745 (7468)
Reflections used for R-free	3864 (378)
R-work	0.1633 (0.1983)
R-free	0.1739 (0.2247)
Number of non-hydrogen atoms	3083
macromolecules	2712
ligands	136
solvent	286
Protein residues	350
RMS(bonds)	0.006
RMS(angles)	0.89
Ramachandran favoured (%)	97.99
Ramachandran allowed (%)	2.01
Ramachandran outliers (%)	0.00
Rotamer outliers (%)	1.03
Clashscore	5.69
Average B-factor	21.10
macromolecules	20.09
ligands	20.28
solvent	30.85

Statistics for the highest-resolution shell are shown in parentheses
